# A Novel Model Based on Necroptosis-Related Genes for Predicting Prognosis of Patients With Prostate Adenocarcinoma

**DOI:** 10.3389/fbioe.2021.814813

**Published:** 2022-01-11

**Authors:** Xin-yu Li, Jian-xiong You, Lu-yu Zhang, Li-xin Su, Xi-tao Yang

**Affiliations:** ^1^ Department of Interventional Radiotherapy, Shanghai Ninth People’s Hospital, Shanghai Jiao Tong University School of Medicine, Shanghai, China; ^2^ Department of Neurosurgery, Shanghai Ninth People’s Hospital, Shanghai JiaoTong University School of Medicine, Shanghai, China; ^3^ Department of Urologic Surgery, The First Affiliated Hospital of Zhengzhou University, Zhengzhou, China

**Keywords:** prostate adenocarcinoma, necroptosis, prognosis, model introduction, cancer

## Abstract

**Background**: Necroptosis is a newly recognized form of cell death. Here, we applied bioinformatics tools to identify necroptosis-related genes using a dataset from The Cancer Genome Atlas (TCGA) database, then constructed a model for prognosis of patients with prostate cancer.

**Methods:** RNA sequence (RNA‐seq) data and clinical information for Prostate adenocarcinoma (PRAD) patients were obtained from the TCGA portal (http://tcga-data.nci.nih.gov/tcga/). We performed comprehensive bioinformatics analyses to identify hub genes as potential prognostic biomarkers in PRAD u followed by establishment and validation of a prognostic model. Next, we assessed the overall prediction performance of the model using receiver operating characteristic (ROC) curves and the area under curve (AUC) of the ROC.

**Results:** A total of 5 necroptosis-related genes, namely *ALOX15, BCL2, IFNA1, PYGL* and *TLR3*, were used to construct a survival prognostic model. The model exhibited excellent performance in the TCGA cohort and validation group and had good prediction accuracy in screening out high-risk prostate cancer patients.

**Conclusion:** We successfully identified necroptosis-related genes and constructed a prognostic model that can accurately predict 1- 3-and 5-years overall survival (OS) rates of PRAD patients. Our riskscore model has provided novel strategy for the prediction of PRAD patients’ prognosis.

## Background Information

Prostate adenocarcinoma (PRAD) is a complex but common malignancy that accounts for about 1300000 new cases and 360,000 deaths every year worldwide. Notably, PRAD accounts for 15% of all new tumor-related cases, making it the second most common neoplasia in elderly males, and the fifth most frequent cause of cancer-related deaths worldwide (Bray et al., 2018; Siegel et al., 2020). In China, PRAD is fast increasing, owing to the rapid socio-economic development in the country, coupled with changes in people’s living and eating habits, as well as an increase in the aging population. Consequently, the disease has become one of the most common urogenital malignancies among elderly Chinese males (Wei et al., 2020). Current treatment options for PRAD are limited, while patient prognosis remains unsatisfactory. Therefore, prospecting for novel prognostic markers is imperative to development of effective treatment strategies and enhanced prognosis of PRAD patients. Previous studies have shown that necroptosis, which was first identified and named in 2005, is regulated by intracellular signaling pathways (Degterev et al., 2005). Notably, this phenomenon can be unmediated by caspases, thus functioning upon inhibition of apoptotic pathways. Moreover, its cellular morphology is consistent with that of conventional necrosis. Additional research evidences have shown that necroptosis is not only an important mechanism underlying cell death, but also plays a crucial role in development and progression of many immune system diseases, including chronic kidney diseases, cerebral ischemia, myocardial ischemia, acute and chronic neurodegenerative diseases, as well as tumors and many other human pathological activities (Jouan-Lanhouet et al., 2014; Barbosa et al., 2018). Although necroptosis exerts important functions in oncogenesis and anticancer processes, only a handful of studies have described its significance in PRAD. In the present study, we systematically analyzed differential expression profiles of necroptosis-related genes between normal and PRAD tissues, then developed a novel risk-score-based model for predicting prognosis of PRAD patients.

## Material and Methods

### Data Acquisition and Differential Gene Expression Analysis

Necroptosis-related genes were extracted from previous studies (Fan et al., 2014; Frank and Vince, 2019; Gong et al., 2019; Yuan et al., 2019; Tang et al., 2020). RNA sequence dataset belonging to 499 PRAD patients and 52 normal controls, together with corresponding clinical information were accessed and downloaded from the TCGA database. Next, we employed the “limma” package implemented in R software to identify differentially expressed genes (DEGs) between the tumor and adjacent normal tissues, based on FDR <0.05 and |log2FC| ≥ 1. Thereafter, we recruited a total of 80 PRAD patients at The First Affiliated Hospital of Zhengzhou University, as the validation cohort. All patients voluntarily signed a written informed consent prior to inclusion in the study, and ethical approval for the study protocol was obtained from The First Affiliated Hospital of Zhengzhou University.

### Construction and Validation of a Prognostic Model

The prognostic value of each DEG was first assessed by univariate Cox regression analysis, then genes that were significantly correlated with OS in PRAD patients identified. To avoid overfitting of the model, we performed Lasso regression analysis to further select significant prognostic genes for OS in PRAD patients, using a penalty parameter tuning (λ) that was conducted by 10-fold cross-validation based on minimum criteria. The identified significantly expressed genes were then incorporated into a multivariate Cox regression model, and the risk score of each patient calculated using the following formula: risk score = esum (each gene’s expression × corresponding coefficient). Median risk-scores were then used to stratify patients into either high- or low-risk groups, and validation of model feasibility and accuracy conducted by generating AUC of the ROC as well as calibration plots. Next, we applied the Cox proportional hazards regression model to analyze these risk factors in PRAD, targeting risk scores, gender, age, as well as the T, N, and M stages. To validate the established model, based on TCGA, we used the median scores to divide patients in the validation cohort into high- and low-risk groups. We also validated the model by stratifying patients in the validation cohort into low- and high-risk subgroups based on the median value of risk scores using the same formula as in the TCGA cohort.

### Validation of Gene Expression

Next, we performed quantitative real-time polymerase chain reaction (qRT-PCR) analysis to quantify expression of DEGs used for model construction in the validation cohort. Briefly, total RNA was extracted from thoroughly ground (under liquid nitrogen) target tissues using the Trizol reagent (Life Technology, Grand Island, NY, United States). The RNA was reverse transcribed to complementary DNA (cDNA) using the RevertAid First Strand cDNA Synthesis Kit (Thermo Scientific, Lithuania), then subjected to qRT-PCR using the SYBR^®^ Green qPCR mix 2.0 kit performed on the Roche LightCycler^®^ 480 Real-Time PCR System. The primers for the genes targeted in this study were obtained from TsingKe Biological Technology (Nanjing, China), and their sequences are as follows: PYGL (forward 5′- CAG​CCT​ATG​GAT​ACG​GCA​TTC -3′, reverse 5′- CGG​TGT​TGG​TGT​GTT​CTA​CTT​T-3′), ALOX15 (forward 5′-GGG​CAA​GGA​GAC​AGA​ACT​CAA-3′, reverse 5′- CAG​CGG​TAA​CAA​GGG​AAC​CT-3′), BCL2 (forward 5′- GGT​GGG​GTC​ATG​TGT​GTG​G -3′, reverse 5′-CGG​TTC​AGG​TAC​TCA​GTC​ATC​C-3′), IFNA1 (forward 5′-GCC​TCG​CCC​TTT​GCT​TTA​CT-3′, reverse 5′-CTG​TGG​GTC​TCA​GGG​AGA​TCA -3′), TLR3 (forward 5′- TTG​CCT​TGT​ATC​TAC​TTT​TGG​GG -3′, reverse 5′-TCA​ACA​CTG​TTA​TGT​TTG​TGG​GT -3′) β-actin (Forward: 5′-CGA​GCA​CAG​AGC​CTC​GCC​TTT​GCC-3′, Reverse: 5′-TGT​CGA​CGA​CGA​GCG​CGG​CGA​TAT-3’). Relative mRNA expression was calculated using the 2-ΔΔCt method.

### Functional Enrichment and Drug Sensitivity Analyses

We first used the “cluster Profiler” package in R to perform Gene Ontology (GO) functional and Kyoto Encyclopedia of Genes and Genomes (KEGG) pathway enrichment analyses on the screened necroptosis-related DEGs (Zeng et al., 2021). Next, we applied the “org.Hs.eg.db” package to identify significantly enriched genes and classify gene clusters. All analyses were performed at a statistical significance of *p* < 0.05. The obtained DEG list was compared with a reference dataset from the CMap database, to obtain a correlation score based on enrichment of DEGs in the reference gene expression profile. A positive number indicated that the DEG was similar to the reference gene expression profile, thus allowed analysis of similar interventions that produce the above effects. Conversely, a negative number indicated that the DEGs had an opposite gene expression profile to that of the reference, suggesting that the drug may antagonize the DEGs’ expression. Ultimately, potentially effective drugs that can counteract drug resistance were inferred from the genetic changes in drug-resistant cell lines, and ranked according to correlation scores obtained on the reference gene expression profile.

### Analysis of Immune Infiltration and Tumor Microenvironment

Considering that immune infiltration levels are correlated with survival and prognosis of cancer patients, we evaluated the relationship between risk-scores and immune infiltration levels. Specifically, we applied multiple algorithms implemented in the IOBR package in R (Zeng et al., 2021) to estimate proportions of tumor-infiltrating immune cells. Next, we calculated stromal and immune scores for each sample using the ESTIMATE algorithm implemented in the ‘limma’ and ‘estimate’ packages in R.

## Results

### Profiles of DEGs

**FIGURE 1:**
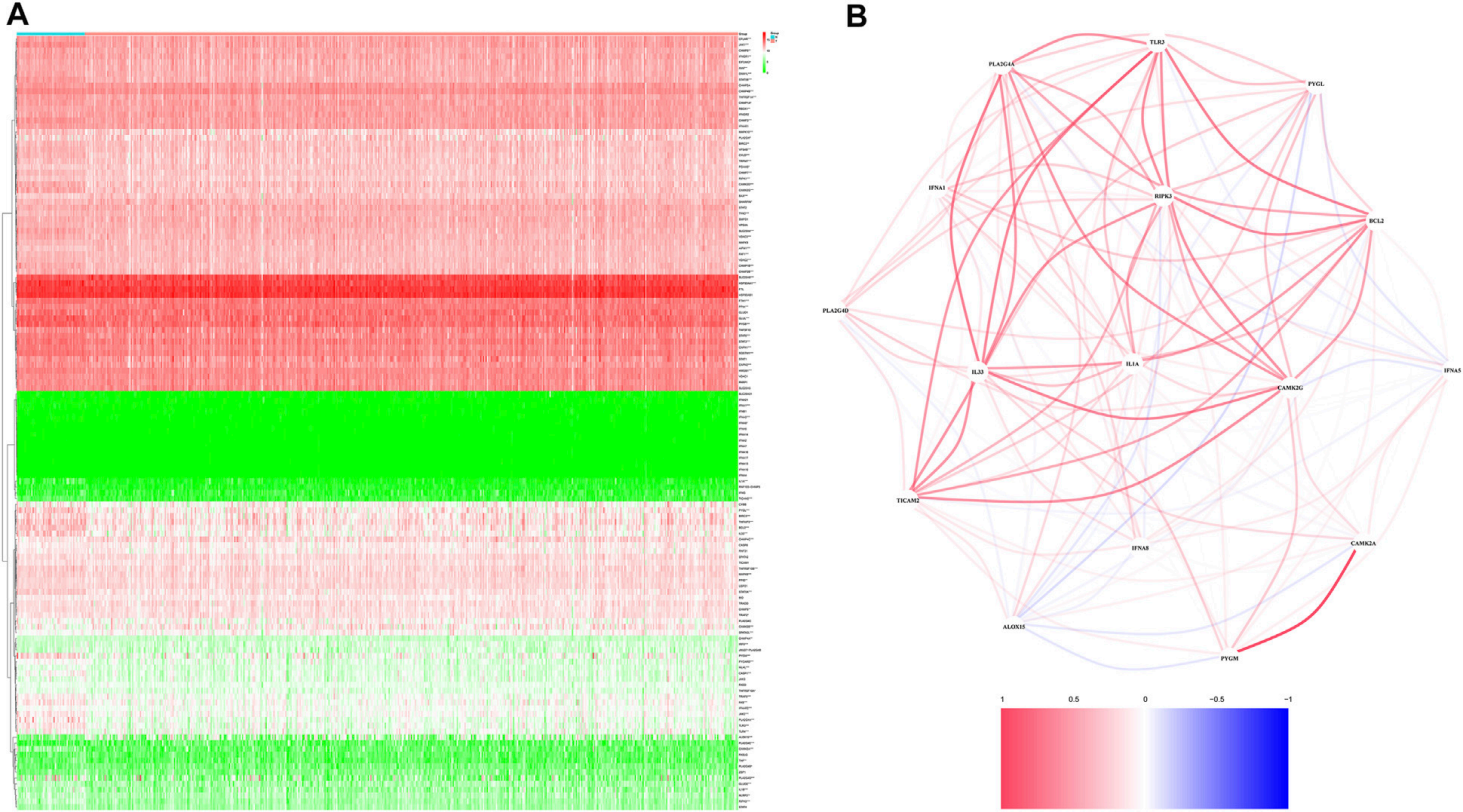
Identification of the candidate genes. **(A)**. Heatmap showing differentially expressed genes between the two groups. **(B)**. The relationship among necroptosis-related DEGs.

**FIGURE 2:**
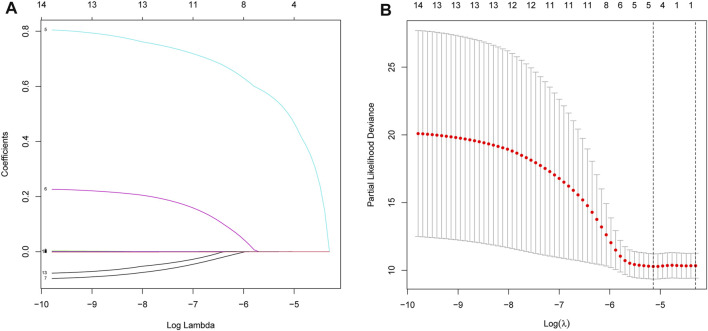
Processes of LASSO Cox model fitting. **(A)**. Profiles of coefficients in the model at varying levels of penalization were plotted against the log(lambda) sequence. **(B)**. Ten-fold cross-validated error (the first and second vertical lines denote the minimum and cross-validated errors, respectively, within 1 standard error of the minimum).

A total of 499 and 80 PRAD patients from the TCGA and validation cohorts, respectively, were included in this study. Detailed clinical characteristics of these patients are shown in Tables 1, 2. A total of 16 necroptosis-related genes were differentially expressed between tumor and adjacent non-tumor tissues. Profiles of these expression patterns are presented using heatmaps in Figure 1A, while the relationships among the DEGs are shown in Figure 1B. Next, we performed LASSO regression analysis to screen covariates, then applied a 10-fold cross validation with minimum criteria to obtain an optimal λ. The final value of λ yielded a minimum cross validation error. Consequently, a total of 5 DEGs, namely *ALOX15, BCL2, IFNA1, PYGL* and *TLR3*, were identified (Figures 2A,B). Incorporation of these significant DEGs into a multivariate Cox regression model revealed coefficients of included each gene. Finally, each PRAD patient was assigned a separate risk score according to the aforementioned formula.

**TABLE 1:** Basic clinical characteristics of PRAD patients in TCGA PRAD cohort.

Characteristic	Levels	Overall
n		499
T stage, n (%)	T2	189 (38.4%)
	T3	292 (59.3%)
	T4	11 (2.2%)
N stage, n (%)	N0	347 (81.5%)
	N1	79 (18.5%)
M stage, n (%)	M0	455 (99.3%)
	M1	3 (0.7%)
Race, n (%)	Asian	12 (2.5%)
	Black or African American	57 (11.8%)
	White	415 (85.7%)
Age, n (%)	≤60	224 (44.9%)
	>60	275 (55.1%)
Age, median (IQR)		61 (56, 66)

**TABLE 2:** Basic clinical characteristics of PRAD patients in validation cohort.

Characteristic	Levels	Overall
n		80
T stage, n (%)	T2	32 (40%)
	T3	40 (50%)
	T4	8 (10%)
N stage, n (%)	N0	64 (80%)
	N1	16 (20%)
M stage, n (%)	M0	72 (90%)
	M1	8 (10%)
Age, n (%)	≤60	37 (46%)
	>60	43 (54%)
OS event, n (%)	Alive	64 (80%)
	Dead	16 (20%)
Age, median (IQR)		59 (52, 69)

### Construction and Validation of a Prognostic Model

**FIGURE 3:**
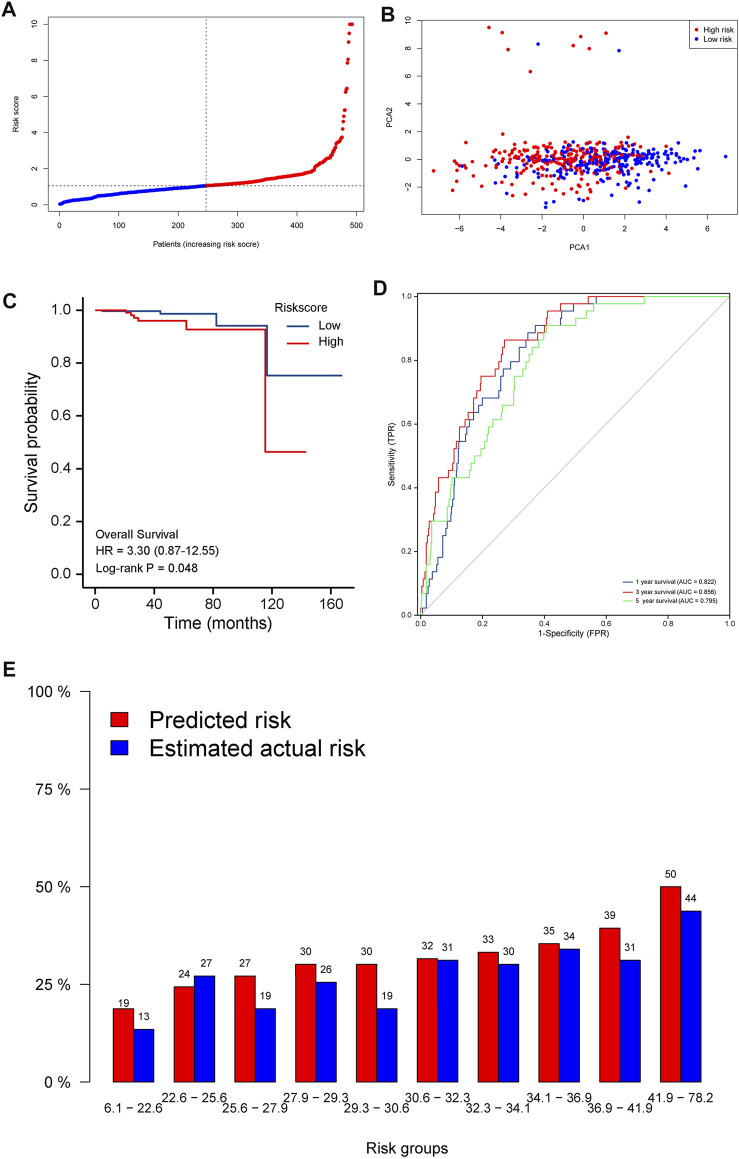
Prognostic value of the 5-gene signature model in the test (TCGA) cohort. **(A)**. Distribution and median values of the risk scores. **(B)**. PCA plot. **(C)**. Kaplan-Meier curves showing OS of patients in the high-risk and low-risk groups. **(D)**. AUC values of the time-dependent ROC curves showing prognostic performance of the risk score. **(E)**. Calibration plots for the established model.

Risk scores = 0.05834^∗^ expression level of *ALOX15*–0.0004914^∗^ expression level of *BCL2*+ 0.665* expression level of *IFNA1*+0.03093^∗^ expression level of *PYGL* −0.0008311^∗^ expression level of *TLR3*. The resulting median cut-off value was used to stratify patients into high-risk and low-risk groups, comprising 249 and 250 individuals, respectively (Figure 3A **)**. A principal component analysis (PCA) plot showed that patients in both groups were distributed in different directions, indicating that the established model had excellent predictive ability to distinguish between high and low-risk PRAD (Figure 3B **)**. Kaplan-Meier curves showed that patients in the low-risk group were significantly associated with higher survival rates compared to their high-risk counterparts (*p* < 0.05) (Figure 3C **)**. In addition, we generated time-dependent ROC curves to estimate performance of the risk prediction model. AUC values for the prognostic model were 0.822, 0.856, and 0.795 for 1-, 3-, and 5-years survival, respectively (Figure 3D **)**. The calibration curve was close to 45°, indicating that the model had good prognostic performance (Figure 3E **)**.

**FIGURE 4:**
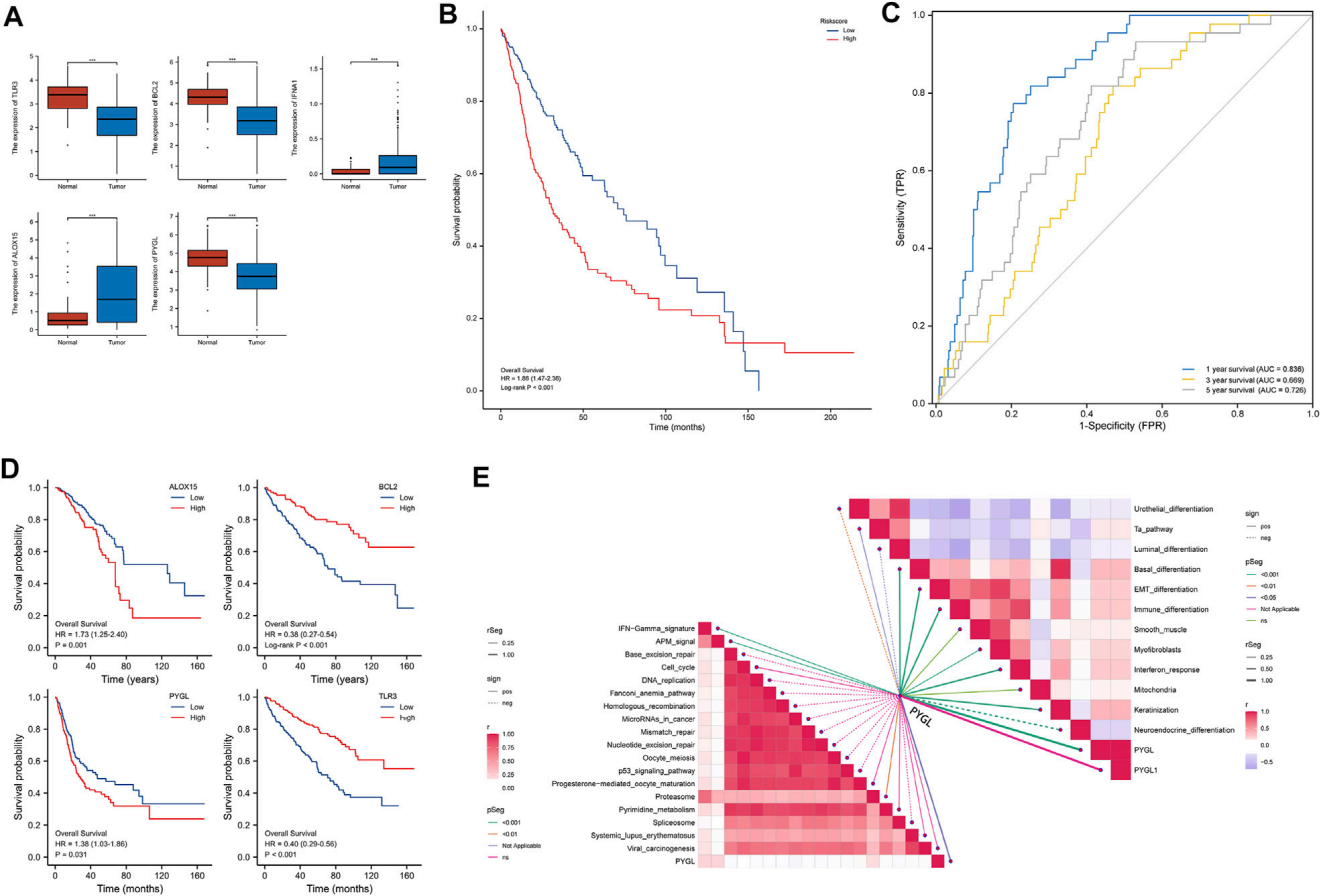
Validation of the 5-gene signature using a validation cohort. **(A)**. Results of qRT-PCR analysis. **(B)**. Kaplan-Meier curves showing OS of patients in the high-risk and low-risk groups. **(C)**. AUCs of time-dependent ROC curves indicating the prognostic performance of the risk score. **(D)**. Kaplan-Meier curves showing OS of patients in the high- and low-expression groups of 5 DEGs. **(E)** Pathway analysis targeting PYGL.

qRT-PCR results showed that *BLC2, PYGL,* and *TLR3* were significantly downregulated in PRAD relative to normal adjacent tissues in the validation cohort (Figure 4A). Results from risk score analysis for each patient, calculated using the same formula and critical values, indicated that patients in the high-risk group had significantly worse overall survival rates than those in the low-risk group (*p* < 0.05) (Figure 4B **)**. AUC values for the 5 necroptosis-related gene signature were 0.836, 0.669, and 0 0.726 at 1, 3 and, 5 years, respectively (Figure 4C **)**. Figure 4D shows the statistically significant results for the genes that make up the model. Multivariate cox regressionreveals that the most significant in 5 DEGs was PYGL. Based on these findings, we further explored the relationship between *PYGL* and multiple pathways, and found that *PYGL* expression was positively correlated with IFN−Gamma signature, APM signaling, Proteasome, Basal differentiation, EMT differentiation, Immune differentiation, Myofibroblasts, Interferon response, and Keratinization, but had a negative correlation with Urothelial differentiation, Luminal differentiation and Neuroendocrine differentiation (Figure 4E **)**. Collectively, these results suggested that *PYGL* expression might be involved in cancer progression.

### Independent Prognostic Value of the Risk Model

Next, we investigated the prognostic significance of different clinical features in PRAD patients. Results from univariate Cox proportional hazard regression analysis revealed that the risk score had prognostic significance in OS (HR = 1.67, 95% CI: 1.23–2.79, Table 3). These results remained significant (HR = 1.42, 95% CI: 1.14–1.75, Table 3) in PRAD patients. Validation of this signature’s prognostic value revealed that it was an independent prognostic factor for PRAD (Table 3). A heatmap of DEGs and distribution of patients’ age as well as survival status across both low- and high-risk subgroups are shown in Supplementary Figure S1.

**TABLE 3:** univariate cox and multivariate cox regression analyses.

Characteristics	Univariate analysis		Multivariate analysis	
	Hazard ratio (95% CI)	*p* value	Hazard ratio (95% CI)	*p* value
TCGA PRAD cohort				
Age (>60 vs. ≤60)	5.28 (3.48–6.86)	<0.001	4.69 (2.36–5.47)	<0.001
Race (Black or African American vs. Asian)	1.74 (0.254–5.84)	0.951	—	—
Riskscore (High vs. Low)	2.29 (1.63–3.65)	<0.001	1.67 (1.23–2.79)	<0.001
T (T1,T2vs. T3,T4)	0.37 (0.18–0.65)	0.041	0.46 (0.24–1.65)	0.21
N(N0vs.N1)	0.62 (0.42–0.81)	0.032	0.72 (0.54–0.89)	0.042
M(M0vs.M1)	0.76 (0.51–1.21)	0.63	—	—
Independent validation cohort				
Age (>60 vs<=60)	4.81 (2.96–5.91)	<0.001	3.51 (2.19–5.78)	0.025
T (T2vs. T3,T4)	0.77 (0.59–0.89)	0.03	0.82 (0.53–0.98)	0.046
N (N0vs.N1)	0.68 (0.31–0.87)	0.02	0.63 (0.25–0.86)	0.031
M (M0vs.M1)	0.87 (0.36–1.86)	0.75	—	—
Riskscore (High vs. Low)	1.87 (1.36–2.05)	<0.001	1.42 (1.14–1.75)	0.034

### High-Risk Scores Were Associated With a Hot Tumor Microenvironment

**FIGURE 5:**
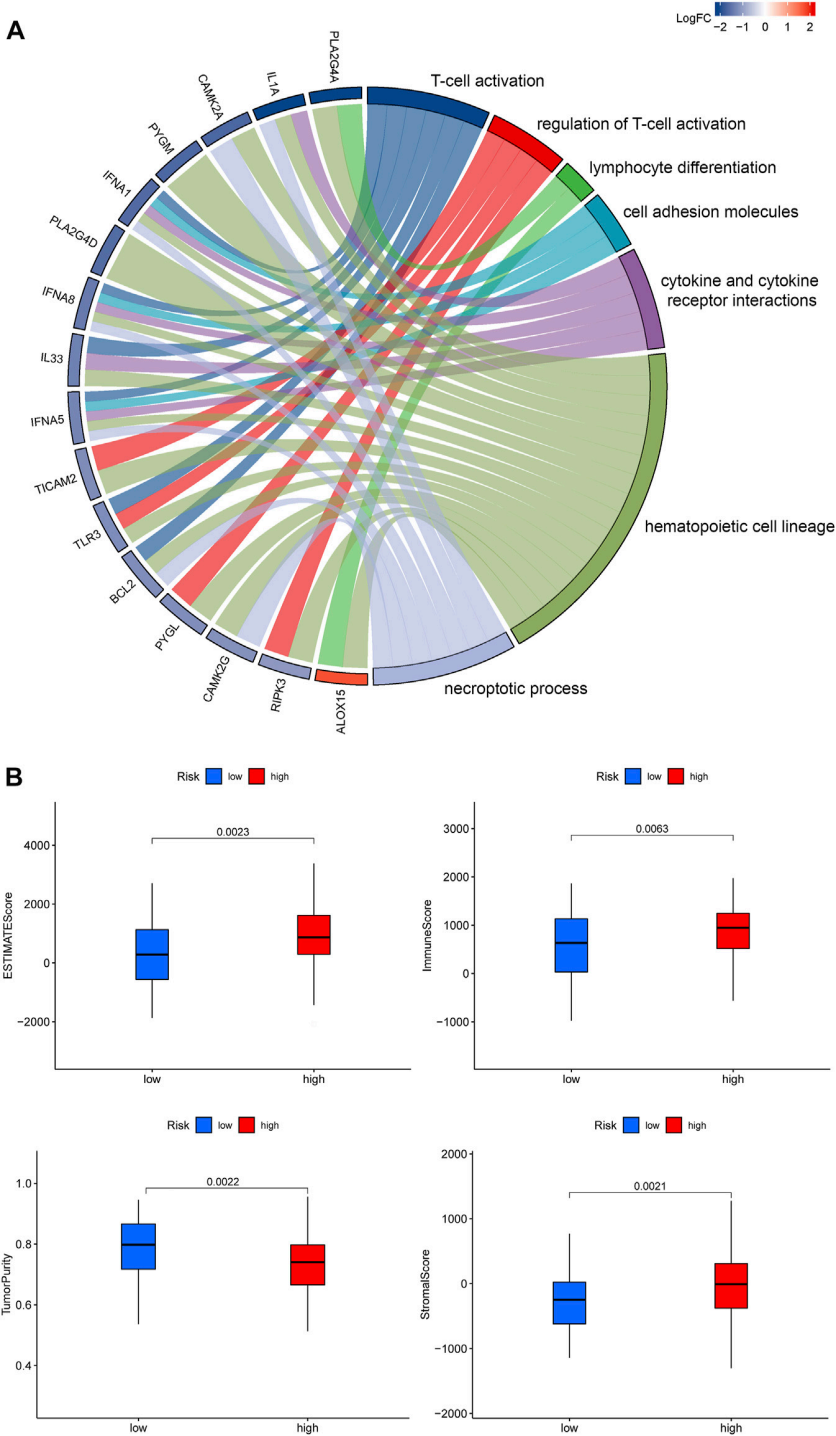
**(A)**. Profiles of GO functional terms and KEGG pathway enrichment for the identified DEGs **(B)**. An overview of the ESTIMATE algorithm.

**FIGURE 6:**
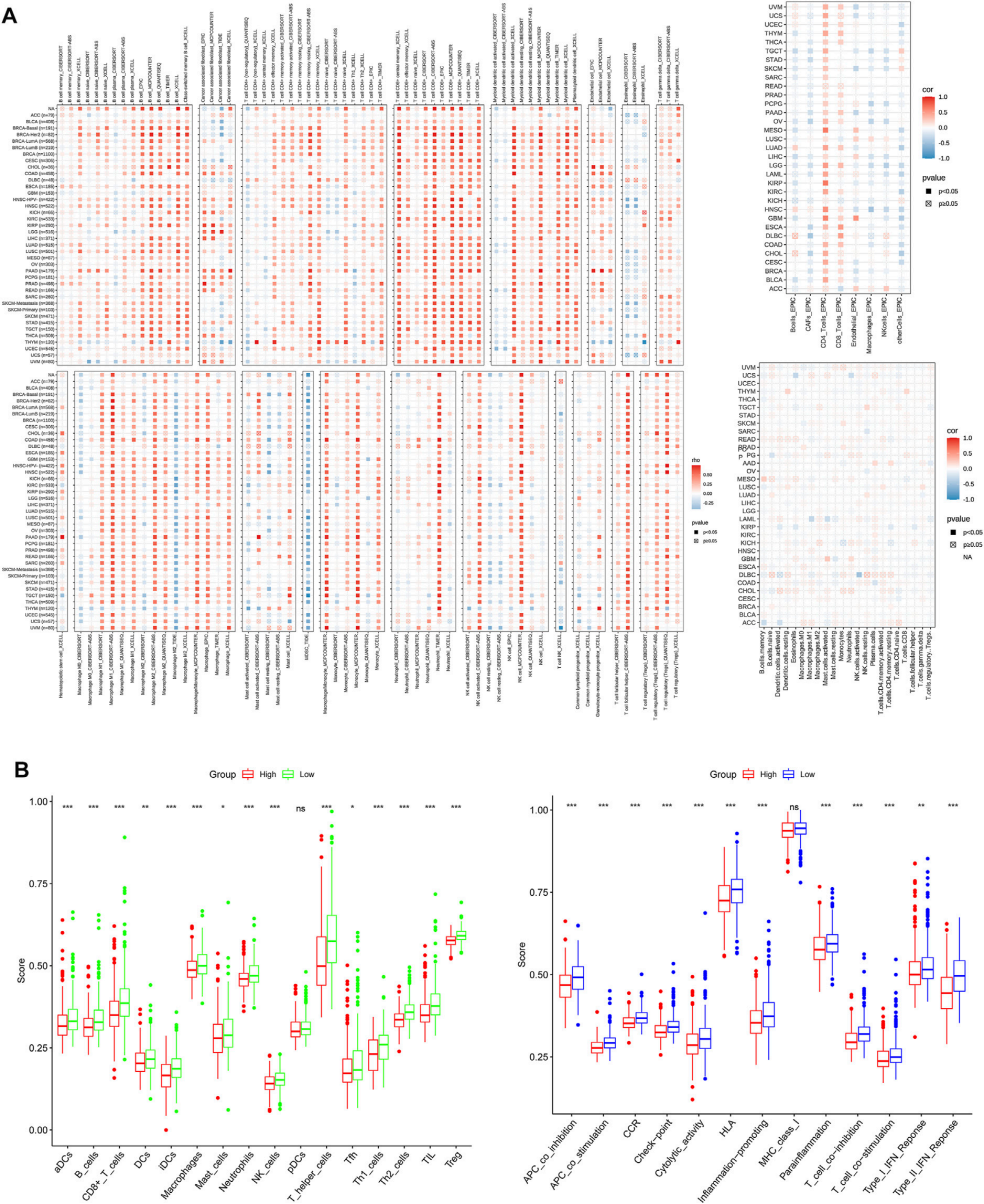
**(A)** The relationship between PYGL expression and immune cell infiltration. **(B)**. Comparison of ssGSEA scores between the two risk groups in the test (TCGA) cohort.

Previous studies have shown that the TME plays a crucial role in tumor extension, progression, migration and invasion (Chalmin et al., 2018). GO terms showed that the DEGs were significantly enriched in immune-related GO entries, including lymphocyte differentiation, and macrophage aggregation. Moreover, KEGG pathway enrichment results revealed that these genes were enriched in cell adhesion molecules, cytokine and cytokine receptor interactions, and necroptosis (Figure 5A). Overall, these results indicated that the DEGs were significantly enriched in immune-related activities, which implies that immune factors represent the main feature of the TME in PRAD. Furthermore, risk scores were positively correlated with the tumor microenvironment (ESTIMATEScore) (Figure 5B). Furthermore, multiple algorithms revealed that *PYGL* expression was strongly associated with macrophages, eosinophils and activated CD4^+^ T cells among others, indicating that the established prognostic model was associated with a hot TME (Figure 6A). ssGSEA results revealed significant enrichment of many types of immune cells, such as macrophages, and Tregs, among others, between the groups, while immune-related functions were also significantly different between the groups (Figure 6B).

### DEG-Based Tumor Classification

**FIGURE 7:**
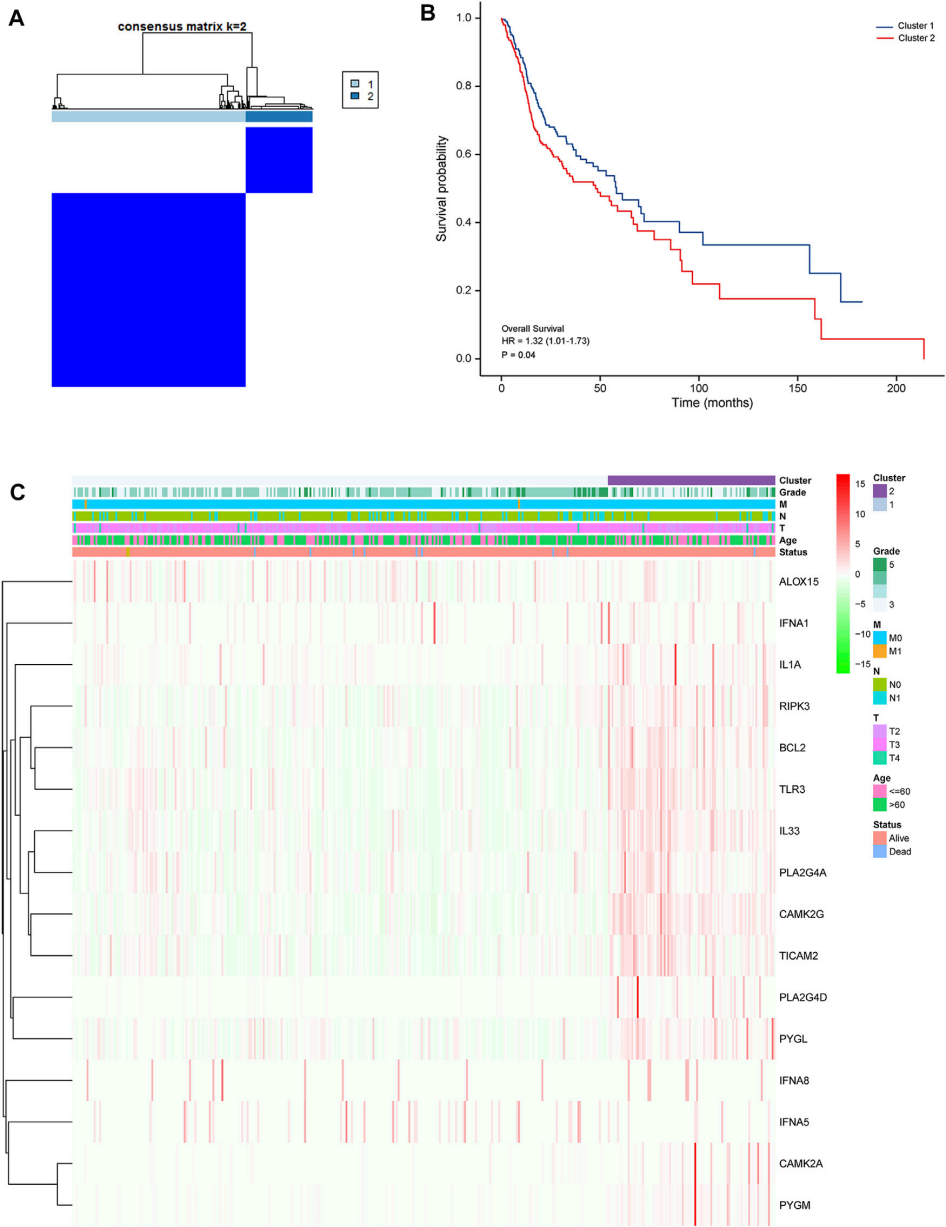
Tumor classification based on the identified pyroptosis-related DEGs **(A)**. Patients were grouped into 2 clusters according to the consensus clustering matrix (k = 2). **(B)**. Kaplan–Meier curves showing OS of patients in the 2 clusters. **(C)**. A heatmap and clinicopathologic characteristics of patients in the 2 clusters based on the identified DEGs.

Next, we performed K-means cluster analysis to stratify PRAD patients into subtypes according necroptosis-related DEGs, and obtained the highest intra- and low inter-group correlations when k = 2 (Figure 7A). Based on results of the 16 DEGs, the 499 PRAD patients were divided into 2 clusters. We generated a heatmap to illustrate the resulting gene expression profiles and found that they were matched to the clinical data (Figure 7B). A comparison of OS revealed significant differences between the clusters (Figure 7C).

### Drug Sensitivity Analysis

**FIGURE 8:**
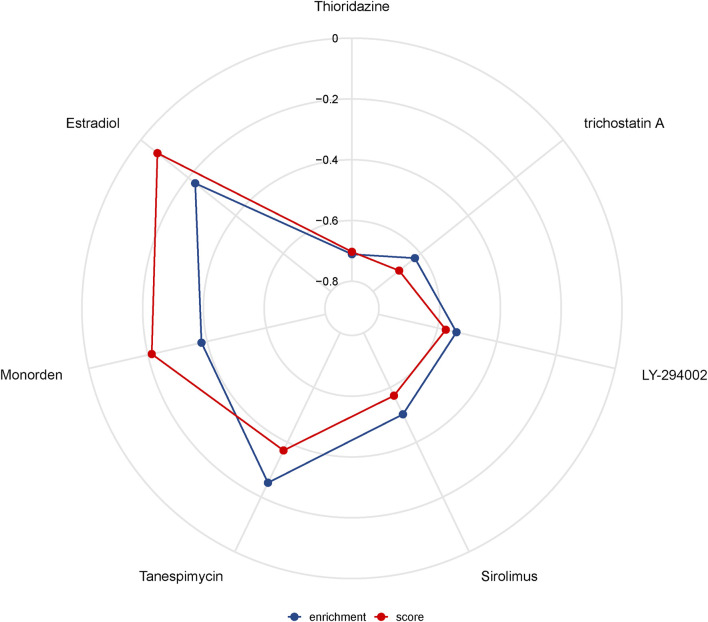
Drug sensitivity analysis.

The highest negative correlation score was obtained in Thioridazine (-0.703), a drug used for treatment of acute schizophrenia, mania and depression. This suggests that this drug has potential therapeutic effects in PRAD. On the other hand, the highest scores were obtained in trichostatin A (an antitumor inhibitor), LY-294002 (the first synthetic protein kinase inhibitor), Sirolimus (an immunosuppressant), Tanespimycin (an antitumor agent), Monorden (an antibiotic), Sirolimus (an immunosuppressant), Tanespimycin (an antitumor agent), and Monorden (an antibiotic). Estradiol (estradiol) is a transdermal estrogenic therapeutic agent that has been used to treat advanced PRAD. The above-mentioned drugs exhibited a strong negative correlation suggesting that they may have potential form treatment of PRAD (Figure 8).

## Discussion

Tumor growth occurs due to an imbalance between tumor cell death and growth (Meier et al., 2000). Notably, inhibition of excessive cell proliferation or normal cell death in the body markedly exacerbates incidence of malignant tumors. Therefore, some researchers believe that unlimited cell proliferation and death inhibition represent the two distinguishing features of malignant tumors. Necroptosis is a newly discovered form of cell death that morphologically manifests with similar characteristics to those observed in necrosis. However, the 2 phenomena differ in that while necrosis is a passive death caused by external physicochemical stresses, such as infection or inflammation, and is not regulated by signaling pathways, necroptosis is a regulated by programmed death. Numerous studies have demonstrated that necroptosis plays an important role in cancer initiation, progression, and metastasis (Jouan-Lanhouet et al., 2014; Barbosa et al., 2018). In the present study, we evaluated the relationship between necroptosis-related genes and prognosis of PRAD patients. Summarily, we screened for key genes that can independently predict prognosis of PRAD patients, then used them to construct a prognostic prediction model. Next, we verified the predictive power of the established model, then applied a multifactorial Cox regression model to assess the effect of other clinicopathological parameters on the signature’s prognostic value in PRAD patients.

A total of 5 DEGs were included in the current model, with ROC and calibration curves in both training and validation cohorts showing that the model had excellent power in predicting PRAD patients. In addition, we selected *PYGL,* a gene located on chromosome 14q22.1 with a total of 20 exons that has been widely used as a building block for predictive models (Luo et al., 2020), and investigated its role in PRAD. Results showed that high *PYGL* expression was an independent predictor of poor prognosis in PRAD patients, consistent with a pervious study that reported similar findings in glioma patients (Luo et al., 2020). Our results further demonstrated that high *PYGL* expression was closely associated with infiltration of immune cells in tumors, with this expression pattern also positively correlated with cancer associated fibroblasts (CAFs). Previous studies have shown that CAFs inhibit T cell infiltration by secreting peritumoral collagen and TGF-Beta/PD-L1 specific antibody YM101, while M7824 could effectively suppress CAFs activity thereby promoting T cell infiltration (Lan et al., 2018; Yi et al., 2021a; Yi et al., 2021b). Additional research evidences have shown that the extracellular matrix produced by CAFs can also limit efficacy of tumor therapy (Ziani et al., 2018; Dou et al., 2020). Therefore, CAFs have become a new target for tumor therapy. For example, inhibition of tumor progression by targeting and regulating CAFs or overcoming their barrier effect has become a new approach for improving efficacy of tumor therapy (Ziani et al., 2018). Since only a handful of reports have described the specific function of *PYGL* in tumorigenesis of PRAD, results of the present study provide new insights into the relationship between necroptosis and PRAD based on *PYGL* functions.

Previous studies have demonstrated the role of the tumor microenvironment, especially the immune microenvironment, in tumor biology (Sumida et al., 2011; Xu et al., 2020; Li et al., 2021). Results of the present study showed that the identified DEGs were mainly enriched in immune-related pathways. Consequently, we performed an immune cell infiltration analysis to assess the relationship between risk scores and overall survival of patients in the context of immune system response. Our results indicated that low-risk patients recruited more immune cells and triggered higher activation of immune pathways than their high-risk counterparts. Notably, the former group exhibited marked enrichment of NK cells. Previous studies have showed that NK cells have a strong cytocidal activity and do not require MHC activation to kill tumor cells (Shu and Cheng, 2020). In addition, tumors have been found to induce production of type II NKT cells, which in turn secrets IL-13, thereby not only causing aggregation of MDSC in the tumor microenvironment but also activating the STAT6 signaling pathway and suppressing CD8^+^ T cell function (Terabe et al., 2000; Terabe et al., 2005). Consequently, the use of NK cells for tumor immunotherapy has gained numerous attention from all over the world. Some of the current NK cell-based clinical approaches for tumor therapy include cytokine-mediated NK cell activation, autologous or allogeneic NK cell transfer, CAR gene-modified NK cells and memory cells. In addition to hematological tumors, modified NK and memory-like NK cells have shown great potential for treatment of liver, non-small cell lung, colorectal, ovarian and breast cancers (Li et al., 2015). Results from our drug sensitivity analysis revealed several potential drugs that might modulate the necroptosis-related genes. Notably, expression of these necroptosis-related genes was negatively correlated with thioridazine, trichostatin A, LY-294002, sirolimus, tanespimycin and monorden, suggesting that these could be potential new options as therapeutic drugs.

## Conclusion

In summary, we identified differentially expressed necroptosis-related genes, between PRAD and normal adjacent tissues, and used them to establish a model for predicting prognosis of PRAD patients. Moreover, we revealed the correlation between risk scores and immune activities. However, further studies are needed to elucidate the mechanisms underlying necroptosis in tumor immunology.

## Data Availability

The datasets presented in this study can be found in online repositories. The names of the repository/repositories and accession number(s) can be found in the article/Supplementary Material.

## Abbreviations

AUC, Area under the curve; APC, Antigen presenting cell; CCR, Cytokine-cytokine receptor; CI, Confidence interval; DEGs, Differentially expressed genes; FDR, False discovery rate; GO, Gene Ontology; HLA, Human leukocyte antigen; HR, Hazard ratio; KEGG, Kyoto Encyclopedia of Genes and Genomes; OS, Overall survival; PRAD, Prostate adenocarcinoma; ROC, Receiver operating characteristic; ssGSEA, Single-sample gene set enrichment analysis; PCA, Principal component analysis; aDC, Activated dendritic cell; iDC, Immature dendritic cell; pDC, Plasmacytoid dendritic cell; TCGA, The Cancer Genome Atlas; Tcm, T central memory; Tfh, T follicular helper cell; TIL, Tumor Infiltrating Lymphocyte; Tgd, T gamma delta.
