# Supplementary figures and images for: A Novel Model Based on Necroptosis-Related Genes for Predicting Prognosis of Patients With Prostate Adenocarcinoma
Source: Front Bioeng Biotechnol. 2022 Jan 11;9:814813. doi: 10.3389/fbioe.2021.814813 (PMC8802148; doi:10.3389/fbioe.2021.814813)

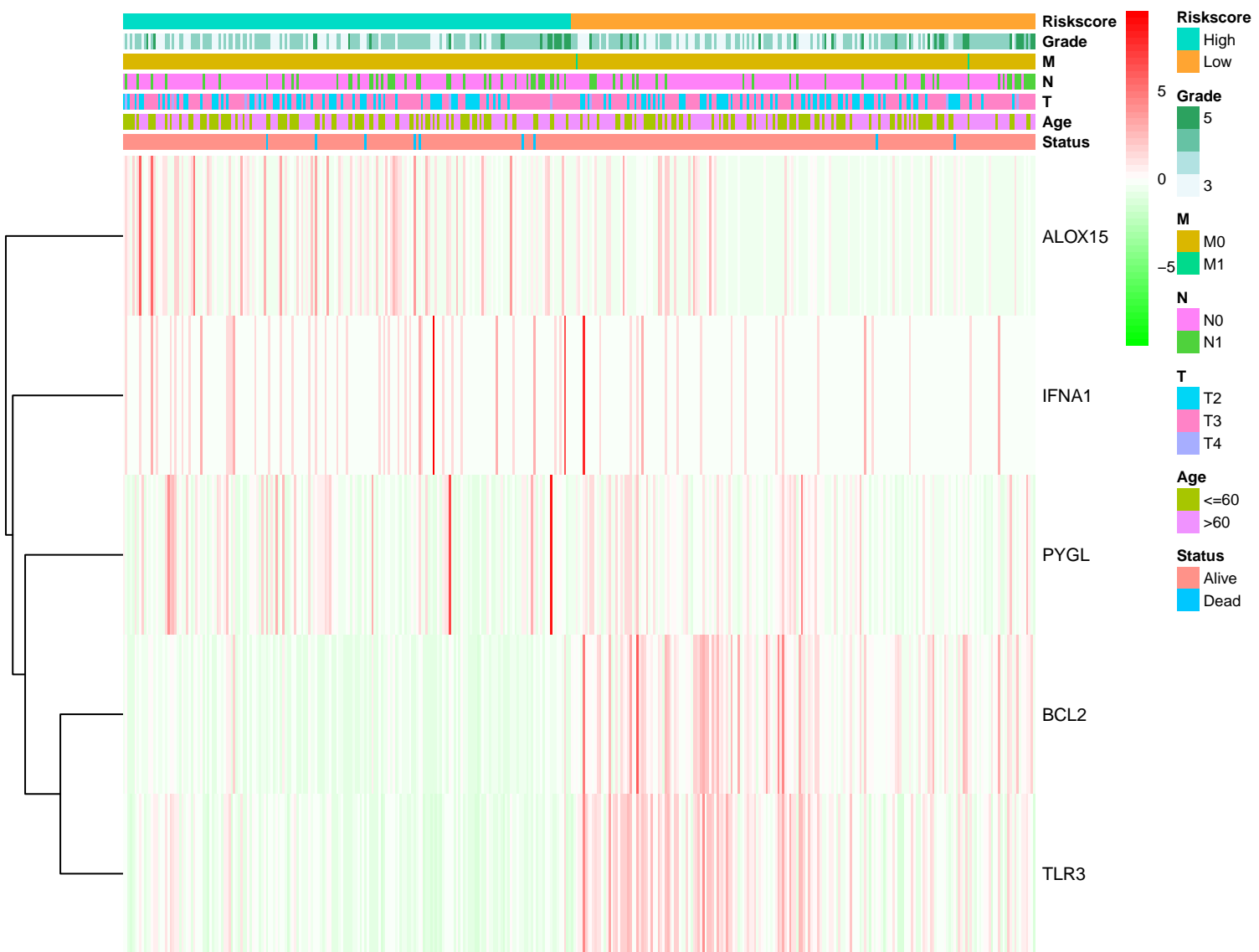

Supplement: Supplementary file 1 [file Image1.pdf]
